# Finger Pulp Infection Due to a Retained Cement Bead: A Report of a Rare Case

**DOI:** 10.7759/cureus.104263

**Published:** 2026-02-25

**Authors:** Aniruddha Sonegaonkar, Satyajit Deshpande, Pallav P Agrawal, Rahul H Sakhare

**Affiliations:** 1 Department of Orthopaedics, NKP Salve Institute of Medical Sciences and Research Centre and Lata Mangeshkar Hospital, Nagpur, IND

**Keywords:** antibiotic, cement spacer, finger injury, pulp necrosis, site of infection

## Abstract

Finger pulp infections commonly occur due to bacteria entering through wounds. Foreign body-related pulp infections are uncommon and can present atypically. In this rare case, infection occurred due to a retained cement bead, which was surgically implanted for treating a compound, comminuted distal phalanx fracture with bone loss. A 22-year-old woman presented with persistent swelling, dull pain, and discharge from the pulp of the right middle finger for three months, unresponsive to empirical antibiotic therapy. Examination revealed localized swelling, tenderness with a puncture wound, and pus discharge from the tip of the right middle finger. The patient gave a history of surgical treatment for a compound fracture of the distal phalanx of the middle finger eight years back with cement bead insertion. X-ray showed a radiopaque oblong shadow embedded in the distal phalanx remnant fragment. Surgical exploration revealed an oblong-shaped cement bead encapsulated within granulation tissue. Removal of this cement bead and drainage resulted in rapid symptom resolution. Retained antibiotic cement beads may cause atypical pulp infections with subtle presentations. It is absolutely necessary to ensure patient follow-up and make the patient understand the need for cement bead removal at the appropriate time to prevent complications.

## Introduction

Infections of the distal digital pulp are commonly caused by *Staphylococcus aureus* following minor skin injuries [1]. These infections, often referred to as felons, usually result from penetrating trauma to the fingertip pulp and may rapidly progress if untreated [2]. However, atypical etiologies-particularly those involving retained foreign bodies-may present with unusual clinical features, leading to delayed diagnosis and prolonged morbidity [3]. Radiographic evaluation plays a crucial role in identifying retained radiopaque foreign materials within the distal phalanx or pulp space [4,5].

Antibiotic-impregnated cement beads are commonly used in orthopedic and hand surgeries as a temporary local drug-delivery system to manage infection and fill dead space, with the intention of planned removal once infection control is achieved. Failure to remove these beads can result in their unintended retention, potentially acting as a foreign body and serving as a source for chronic inflammation or delayed infection. Here, we report a rare case of a finger pulp infection caused by a retained cement bead inserted surgically, resulting in delayed infection several years after the initial procedure.

## Case presentation

A 22-year-old right-hand-dominant woman presented with persistent swelling, dull pain, and discharge from the pulp of the right middle finger, unresponsive to empirical antibiotic therapy. Examination revealed localized swelling, tenderness with a puncture wound, and pus discharge from the tip of the right middle finger for one month. The patient gave a history of surgical treatment for a compound fracture of the distal phalanx of the middle finger eight years back with cement bead insertion. After healing of the surgical wound, the patient did not keep follow-up with the treating surgeon. The patient did not have any significant symptoms for nearly eight years, until three months back, when she developed pain, swelling, and eventually pus discharge.

Clinical findings

**Figure 1:**
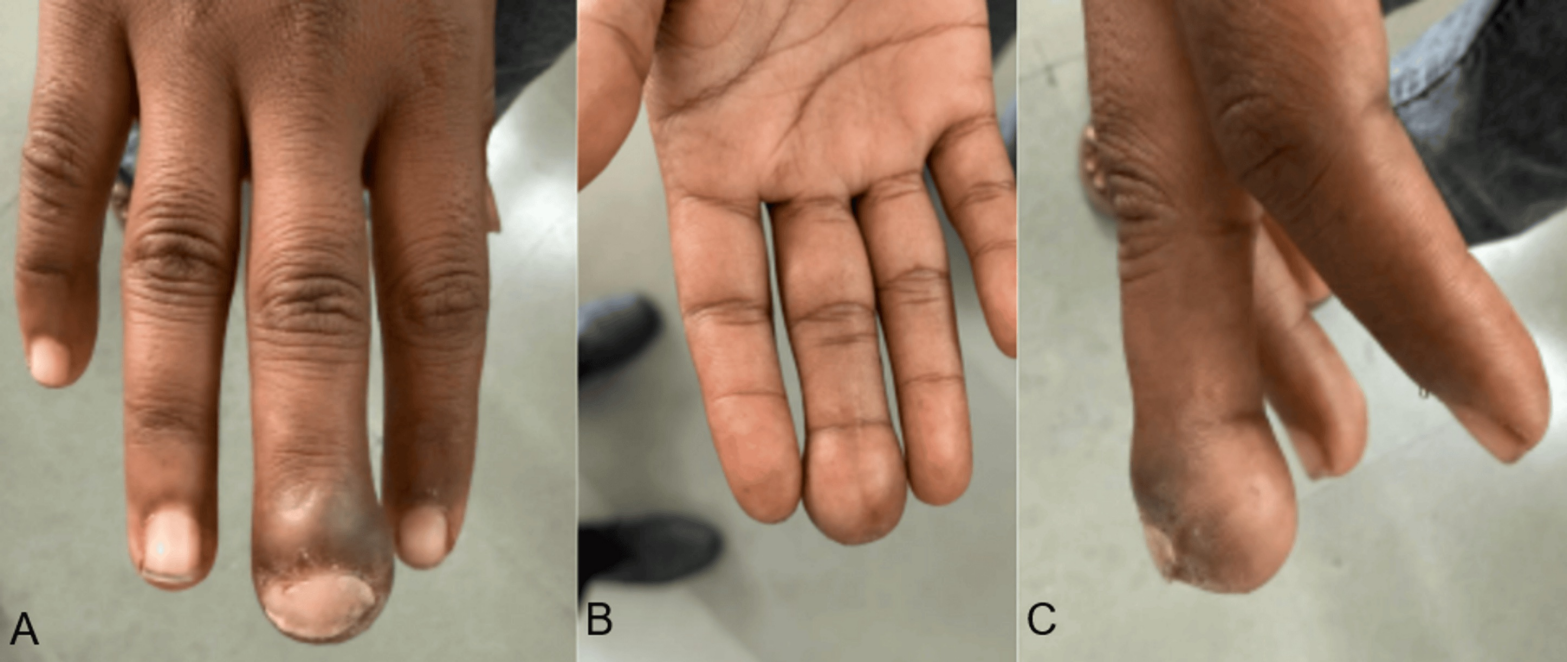
(A-C) Clinical image of the affected middle finger

On examination, there was diffuse swelling of the finger pulp, discharging pus, localized tenderness, full range of motion of the distal interphalangeal joint, intact perfusion, and preserved sensation over the tip of the finger (Figure 1).

Diagnostic assessment

**Figure 2:**
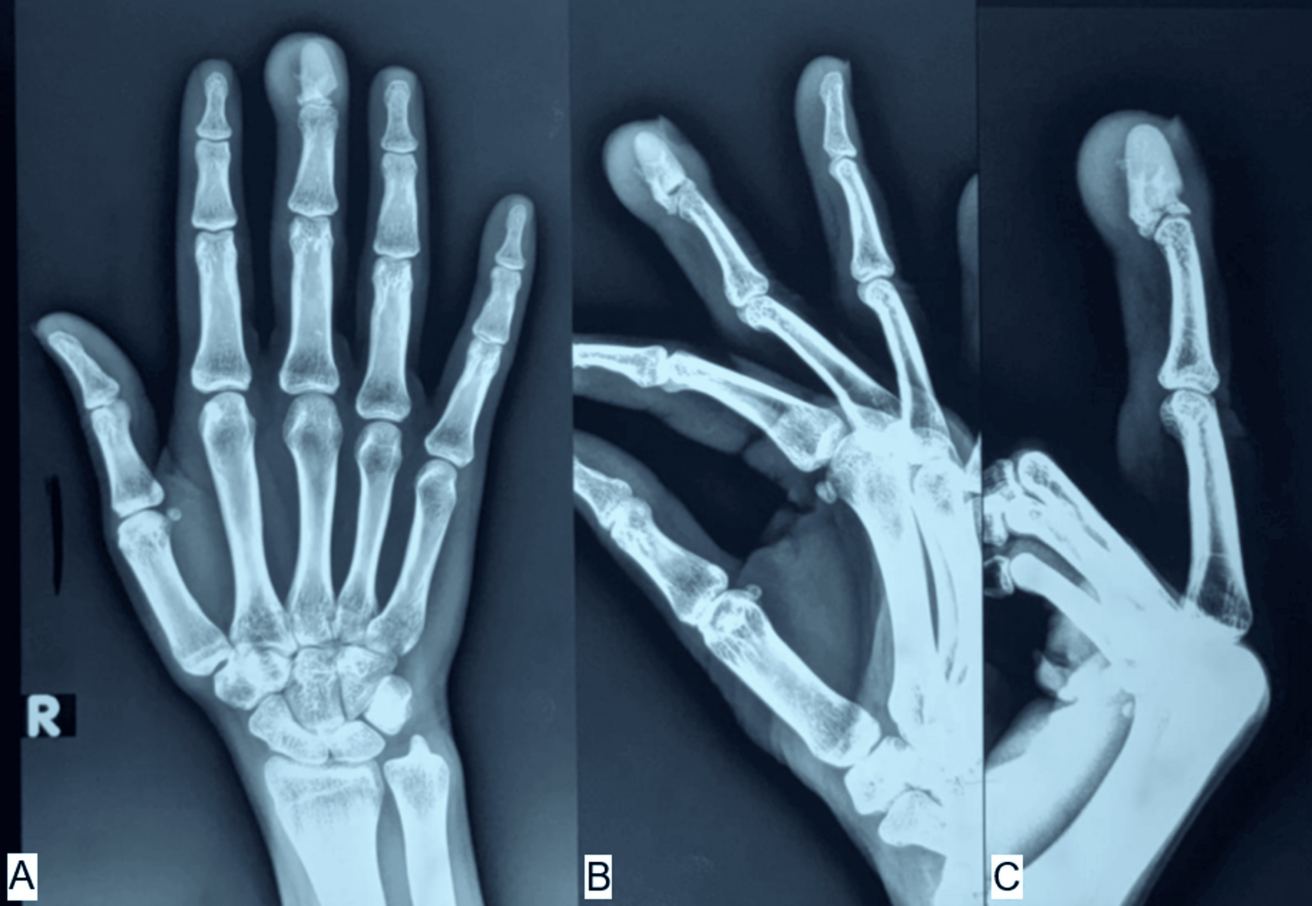
(A-C) X-ray shows an oblong-shaped radiopaque shadow of the cement bead at the tip of the middle finger

Plain radiographs demonstrated soft-tissue swelling with a radiopaque oblong foreign body embedded in the remnant distal phalanx fragment [4]. Retained foreign bodies may remain clinically silent for prolonged periods and later present with chronic infection or discharging sinus, and imaging plays a key role in their detection [5] (Figure 2).

Therapeutic intervention

Under digital block anesthesia, an incision was made from the medial to lateral side on the volar aspect of the finger pulp just proximal to the nail bed, incorporating the sinus tract. Careful dissection of the pulp compartments was performed. A cement bead measuring approximately 14 mm was found encapsulated within granulation tissue. It was gradually separated from the surrounding soft tissues and removed. Soft tissue and discharge samples were sent for culture. The wound was irrigated with betadine, hydrogen peroxide, and normal saline and loosely closed. Postoperatively, the patient received oral clindamycin for seven days.

**Figure 3:**
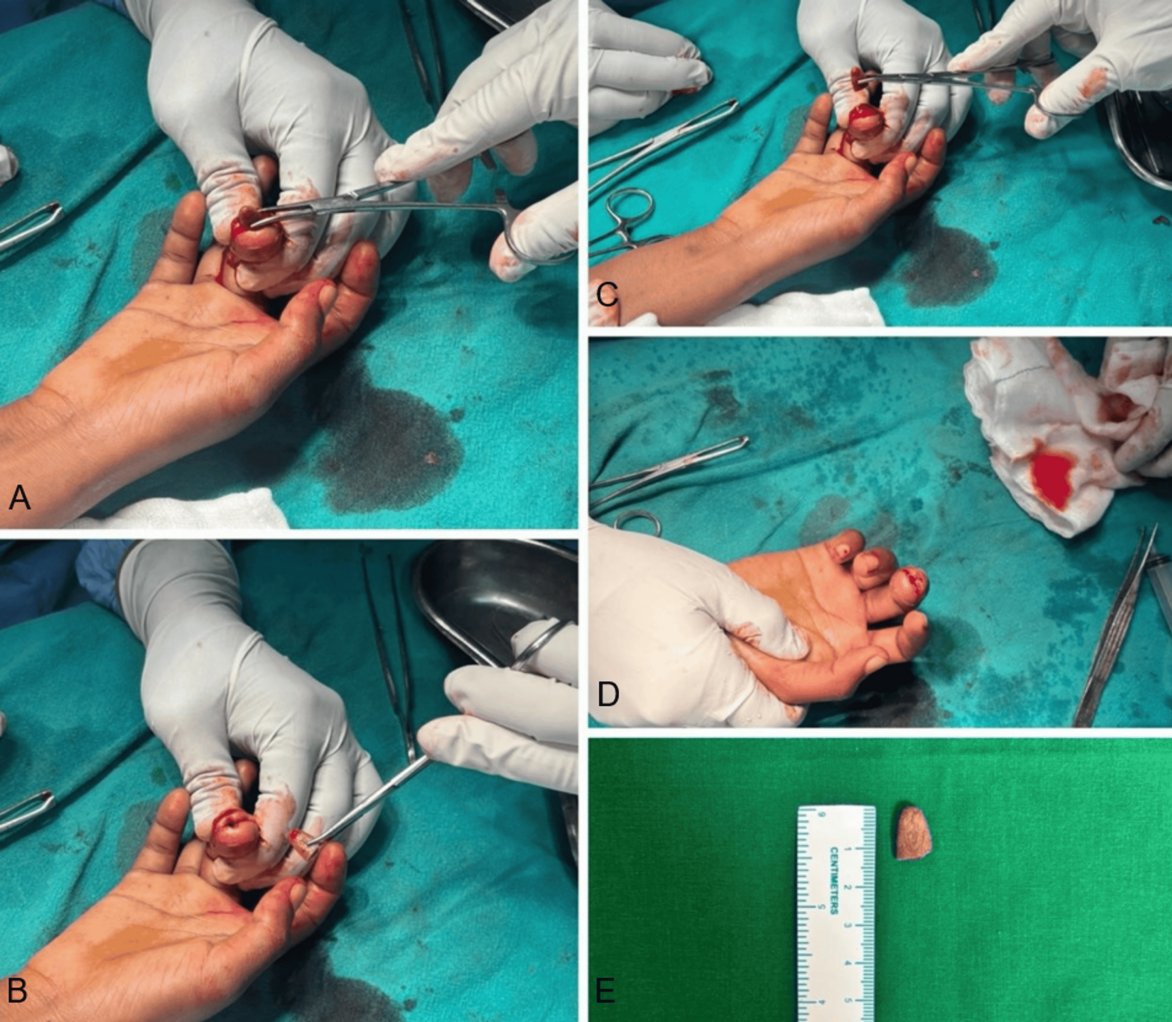
(A-E) Intra-operative pictures and a picture of the explanted cement bead of size 14 mm approximately

**Figure 4:**
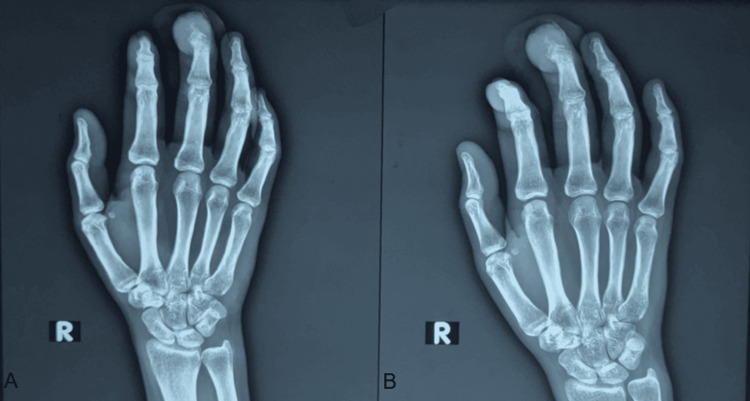
(A, B) Postoperative X-ray

Surgical removal of retained foreign material is essential, as conservative treatment alone is unlikely to resolve infection when an irritant remains in situ (Figures 3, 4) [2,6].

Follow-up and outcomes

**Figure 5:**
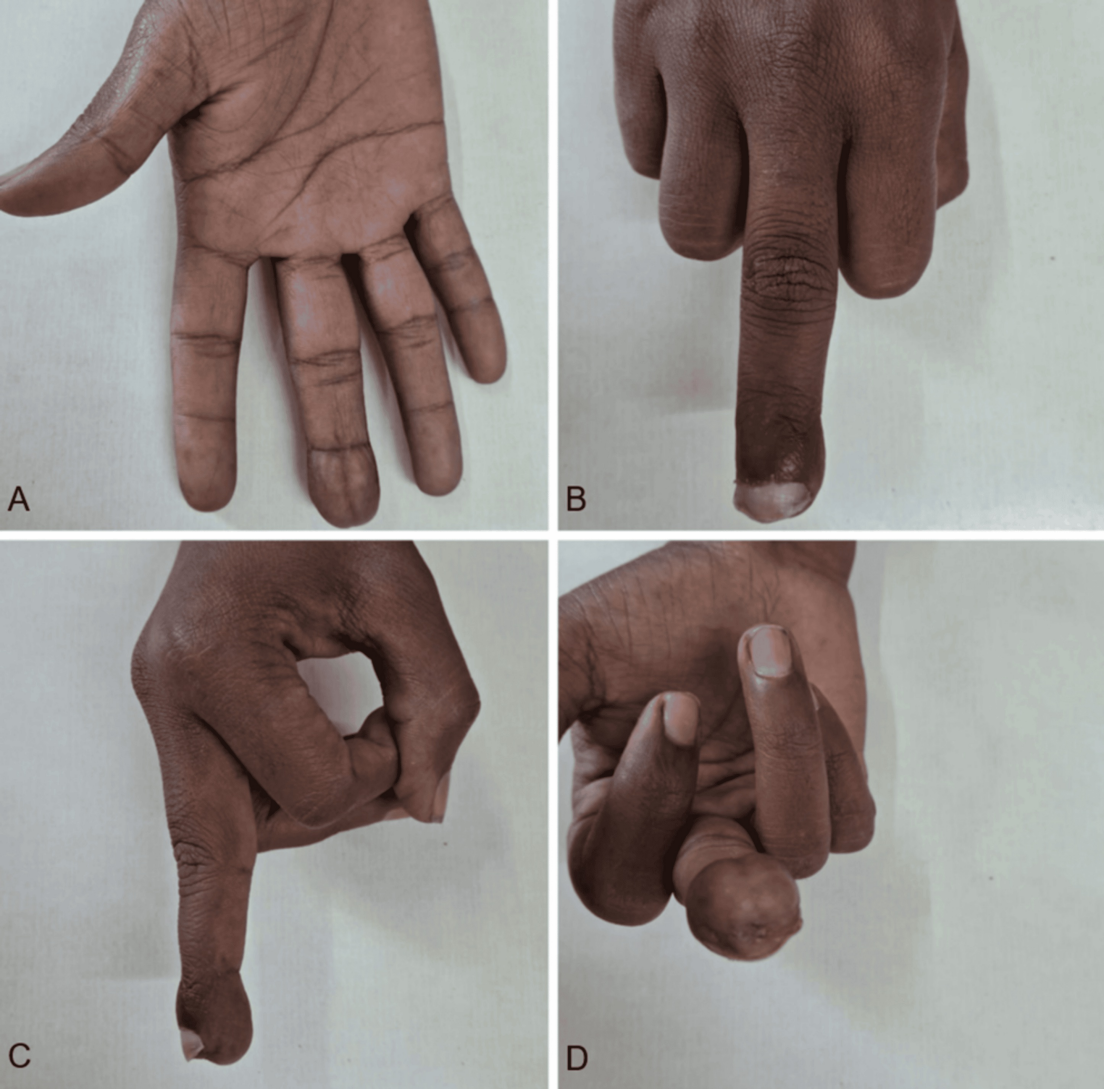
(A-D) Clinical follow-up picture after 6 weeks shows a well-healed wound and no infection

By postoperative day five, pain and swelling had markedly improved. At the two-week follow-up, the wound had healed without complications; the patient regained a cosmetically acceptable, pain-free finger; due to bone loss of the distal phalanx, the fingertip remained soft; and no recurrence of infection was observed (Figure 5).

## Discussion

Although pulp infections are common, those caused by retained foreign materials-especially non-metallic substances-are rare and frequently present atypically [3]. Polymethylmethacrylate (PMMA) bone cement is widely used in orthopedic surgery for fracture management and local antibiotic delivery [6,7]. However, when retained unintentionally, PMMA may act as a chemical irritant, inducing foreign-body granuloma formation and serving as a nidus for secondary bacterial infection [7].

Delayed presentations of hand infections related to retained foreign bodies have been reported, sometimes occurring many years after the initial injury or surgery [3,5]. In the present case, loss of follow-up and failure to remove the cement bead resulted in delayed infection. This highlights the importance of regular postoperative surveillance and thorough patient counseling regarding the need for planned removal of temporary implants [6,7].

## Conclusions

Antibiotic cement beads can become a source of infection if retained after complete antibiotic elution. It is the responsibility of the treating surgeon to ensure that patients understand the importance of timely bead removal and adhere to regular follow-up to prevent delayed complications.

## References

[1] Altman DT, Lubahn JD, Kuhn PJ (1994). A case report and review of mycetoma of the hand: a diagnostic and therapeutic challenge. J Hand Surg Am.

[2] Mohanty SP, Kumar MN, Murthy NS (2003). Use of antibiotic-loaded polymethyl methacrylate beads in the management of musculoskeletal sepsis--a retrospective study. J Orthop Surg (Hong Kong).

[3] Prashant N, Azuhairy A (2018). Actinomycosis of distal phalanx twenty years after flap reconstruction of index finger: a case report. Malays Orthop J.

[4] Heffernan EJ, Alkubaidan FO, White LM, Masri BA, Munk PL (2007). The radiology of antibiotic-impregnated cement. AJR Am J Roentgenol.

[5] Peterson JJ, Bancroft LW, Kransdorf MJ (2002). Wooden foreign bodies: imaging appearance. AJR Am J Roentgenol.

[6] van Vugt TA, Arts JJ, Geurts JA (2019). Antibiotic-loaded polymethylmethacrylate beads and spacers in treatment of orthopedic infections and the role of biofilm formation. Front Microbiol.

[7] Anagnostakos K, Kelm J (2009). Enhancement of antibiotic elution from acrylic bone cement. J Biomed Mater Res B Appl Biomater.

